# Supplementary material for: Evaluation of Climate Change Impacts on the Potential Distribution of Wild Radish in East Asia
Source: Plants (Basel). 2023 Sep 6;12(18):3187. doi: 10.3390/plants12183187 (PMC10534784; doi:10.3390/plants12183187)
Supplement: Supplementary file 1 [file plants-12-03187-s001.zip › Table S1.pdf]

Table 1 Estimates of relative contributions and permutation importance of the predictor environmental variables to the MaxEnt model

Time period	Variable	bio02	bio03	bio05	bio06	bio07	bio08	bio09	bio14	bio15	bio16
LGM	Percent contribution	2.34	0.95	9.44	7.49	1.29	1.45	6.64	56.05	7.51	6.84
	Permutation importance	1.08	1.92	4.09	24.23	6.06	0.53	7.86	4.34	22.11	27.78
Current	Percent contribution	3.58	0.45	3.90	11.45	5.35	1.80	1.05	62.18	8.58	1.67
	Permutation importance	2.19	0.30	0.41	36.84	29.60	0.50	13.50	10.16	5.45	1.06
2070(SSP1-2.6)	Percent contribution	2.77	0.54	5.68	12.07	3.19	1.44	1.90	59.81	10.63	1.97
	Permutation importance	3.43	0.48	1.06	30.01	23.16	0.51	13.98	3.16	21.68	2.53
2070(SSP5-8.5)	Percent contribution	3.98	1.77	2.91	11.90	1.60	4.64	6.03	60.73	6.22	0.21
	Permutation importance	2.15	1.37	0.47	48.75	4.81	0.87	13.98	25.12	1.09	1.40
