# Supplementary figures and images for: Evaluation of Climate Change Impacts on the Potential Distribution of Wild Radish in East Asia
Source: Plants (Basel). 2023 Sep 6;12(18):3187. doi: 10.3390/plants12183187 (PMC10534784; doi:10.3390/plants12183187)

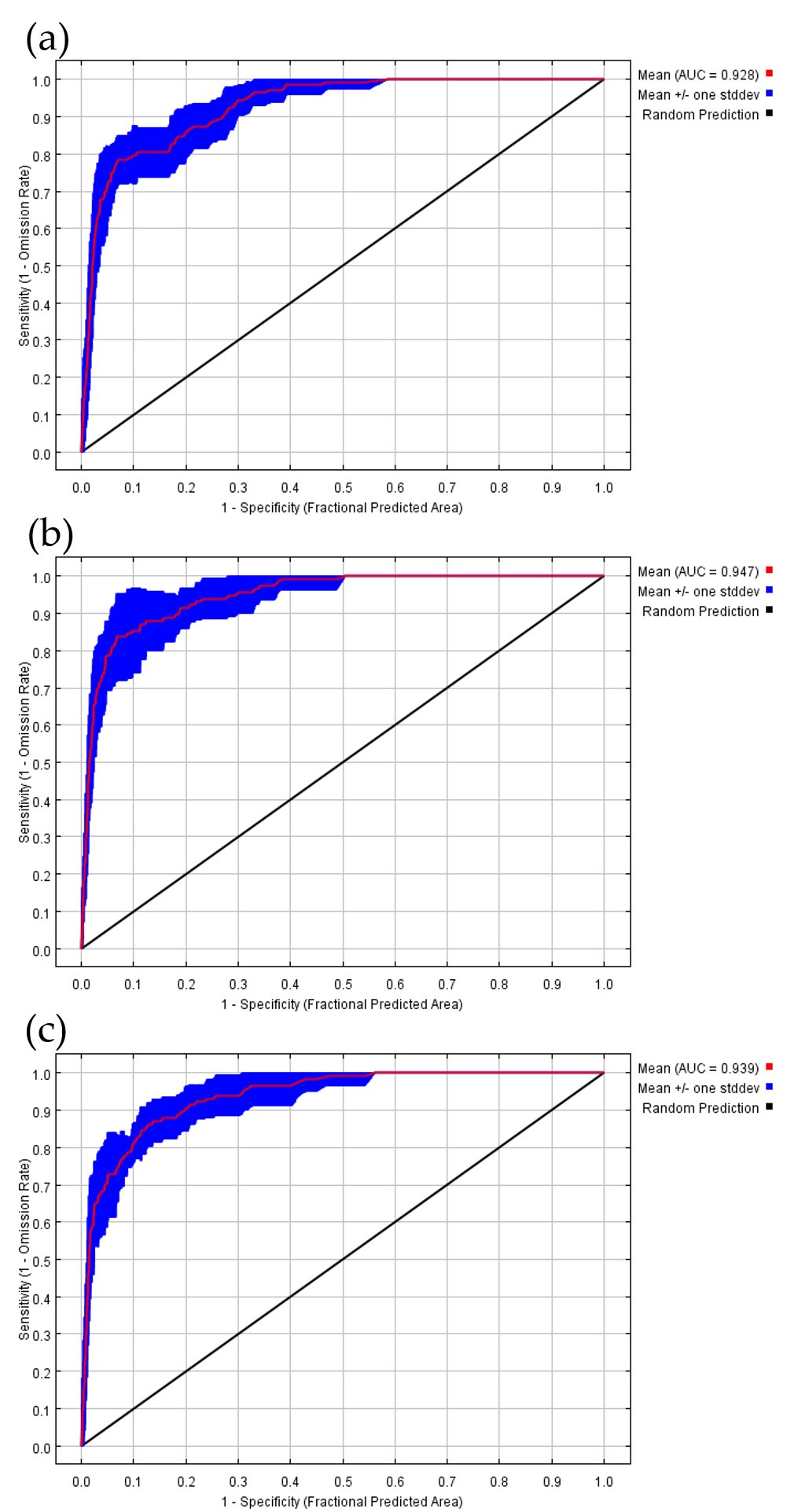

Supplement: Supplementary file 1 [file plants-12-03187-s001.zip › Figure S1.tif]

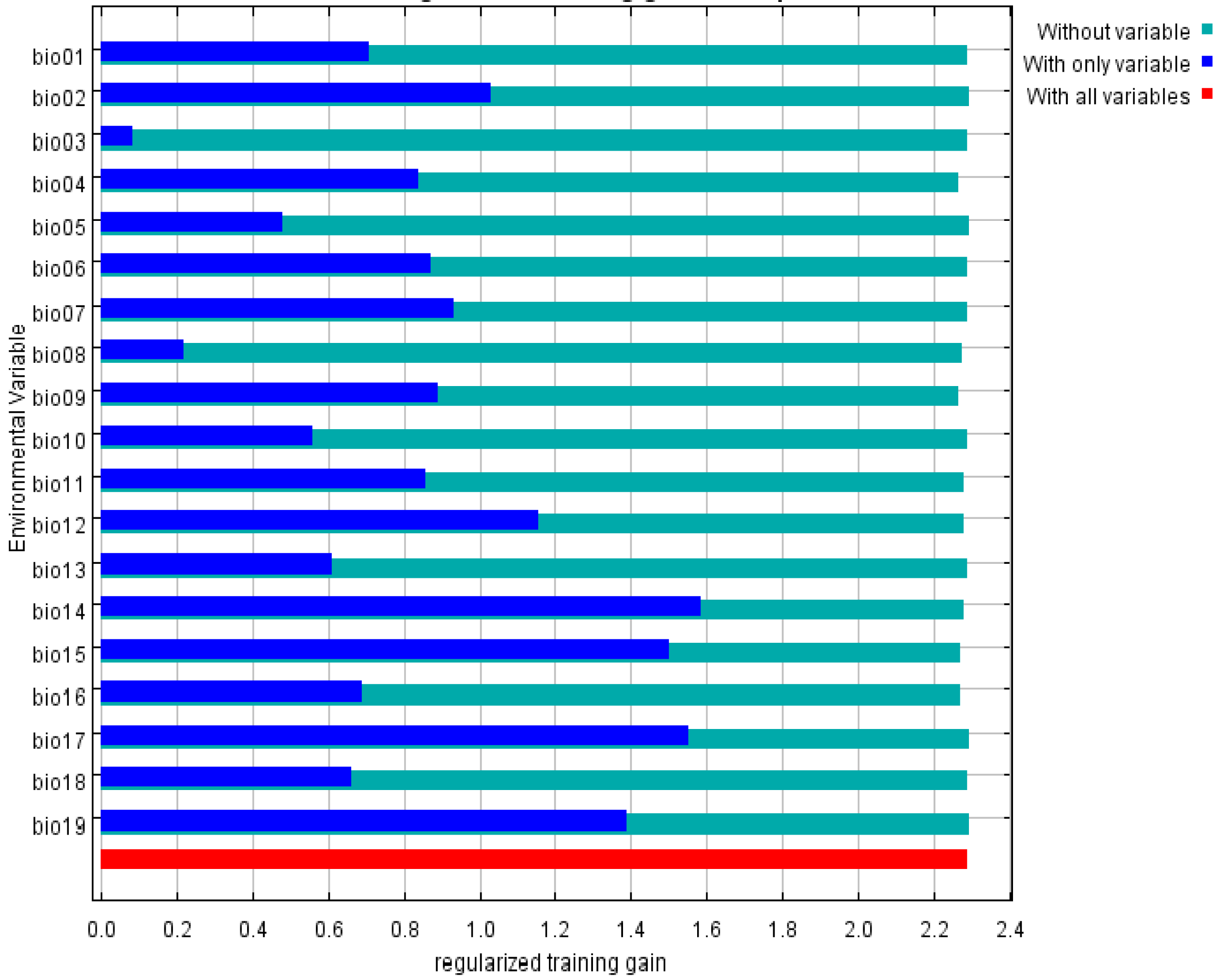

Supplement: Supplementary file 1 [file plants-12-03187-s001.zip › Figure S2.tif]
